# Joint and tendon subclinical involvement suggestive of gouty arthritis in asymptomatic hyperuricemia: an ultrasound controlled study

**DOI:** 10.1186/ar3223

**Published:** 2011-01-17

**Authors:** Carlos Pineda, Luis M Amezcua-Guerra, Carla Solano, Pedro Rodriguez-Henríquez, Cristina Hernández-Díaz, Angelica Vargas, Fritz Hofmann, Marwin Gutiérrez

**Affiliations:** 1 Biomedical Research Subdirection, Instituto Nacional de Rehabilitación, Av. México-Xochimilco 289, Arenal de Guadalupe, Tlalpan, Mexico City 14389, Mexico; 2 Department of Immunology, Instituto Nacional de Cardiología Ignacio Chávez, Juan Badiano 1, Sección XVI, Tlalpan, Mexico City 14080, Mexico; 3 Department of Rheumatology, Hospital Nacional Rosales and Instituto Salvadoreño del Seguro Social, Final calle Arce 25 Av. Norte, San Salvador, El Salvador; 4 Department of Rheumatology, Hospital General Dr. Manuel Gea González, Calzada de Tlalpan 4800, Sección XVI, Tlalpan, Mexico City 14080, Mexico; 5 Department of Musculoskeletal Ultrasonography, Instituto Nacional de Rehabilitación, Av. México-Xochimilco 289, Arenal de Guadalupe, Tlalpan, Mexico City 14389, Mexico; 6 Department of Rheumatology, Instituto Nacional de Cardiología Ignacio Chávez, Juan Badiano 1, Sección XVI, Tlalpan, Mexico City 14080, Mexico; 7 Clinica Reumatologica, Università Politecnica delle Marche, Via dei Colli 52, Jesi, Ancona I-60035, Italy

## Abstract

**Introduction:**

In this study, we aimed to investigate ultrasonographic (US) changes suggestive of gouty arthritis in the hyaline cartilage, joints and tendons from asymptomatic individuals with hyperuricemia.

**Methods:**

We conducted a cross-sectional, controlled study including US examinations of the knees and first metatarsal-phalangeal joints (first MTPJs), as well as of the tendons and enthesis of the lower limbs. Differences were estimated by χ^2 ^or unpaired *t*-tests as appropriate. Associations were calculated using the Spearman's correlation coefficient rank test.

**Results:**

Fifty asymptomatic individuals with hyperuricemia and 52 normouricemic subjects were included. Hyperechoic enhancement of the superficial margin of the hyaline cartilage (double contour sign) was found in 25% of the first MTPJs from hyperuricemic individuals, in contrast to none in the control group (*P *< 0.0001). Similar results were found on the femoral cartilage (17% versus 0; *P *< 0.0001). Patellar enthesopathy (12% versus 2.9%; *P *= 0.01) and tophi (6% versus 0; *P *= 0.01) as well as Achilles enthesopathy (15% versus 1.9%; *P *= 0.0007) were more frequent in hyperuricemic than in normouricemic individuals. Intra-articular tophi were found in eight hyperuricemic individuals but in none of the normouricemic subjects (*P *= 0.003).

**Conclusions:**

These data demonstrate that morphostructural changes suggestive of gouty arthritis induced by chronic hyperuricemia frequently occur in both intra- and extra-articular structures of clinically asymptomatic individuals.

## Introduction

Serum urate (SU) concentration represents the balance between the breakdown of purines and the rate of uric acid renal excretion. Its solubility threshold is approximately 7 mg/dL, and when exceeded, interstitial fluids become oversaturated, which in turn increases the likelihood of monosodium urate (MSU) crystal tissue deposition [1]. The MSU crystal deposition can be clinically expressed as gouty arthritis, tophi formation, urate nephropathy or urolithiasis [2].

While the usefulness of urate-lowering treatment in patients with clinical manifestations of hyperuricemia such as gouty arthritis or nephropathy has been largely established, its use in asymptomatic hyperuricemic individuals is still the object of several controversies [3]. This could in part be related to the limited evidence about the subclinical musculoskeletal involvement in asymptomatic individuals with hyperuricemia [4].

Although the best imaging method to investigate the presence of MSU crystal deposits in the early stages has not yet been established [5], ultrasound (US) has been demonstrated to be a valid imaging modality to detect musculoskeletal involvement in patients with gout [6-9]. The main US findings related to MSU crystal deposition include hyperechoic enhancement of the superficial margin of the hyaline cartilage (double contour sign), hyperechoic spots within tendons and soft tissues, tophi and bone erosions [7,10,11]. Additionally, an increase of blood flow surrounding the MSU deposits detected by power Doppler (PD) has been described as an indicator of inflammatory activity [5,7].

In erosive arthritides, musculoskeletal US has been shown to be a valuable imaging method to confirm early structural damage [12]. Moreover, both magnetic resonance imaging and US are useful advanced imaging techniques to demonstrate occult destructive arthropathy in patients with gout and normal plain radiographs [13]. However, to date, there is only a single US study demonstrating the existence of tophaceous deposits in asymptomatic hyperuricemic subjects; remarkably, that study was focused on the assessment of tendons and synovium pathology of knees and ankles [5]. On the basis of the understanding that MSU tissue deposits occur at multiple sites (especially on the hyaline cartilage), the present study was aimed at investigating the existence of subclinical musculoskeletal involvement in the hyaline cartilage, tendons, soft tissues and lower-limb joints (knees, ankles and first metatarsal-phalangeal joints (first MTPJs)) from asymptomatic individuals with hyperuricemia by means of US.

## Materials and methods

### Patients

The study was performed on 50 consecutive patients with SU concentrations ≥7.0 mg/dL on at least two occasions within the past 2 years who had attended the outpatient clinics of the rheumatology, cardiology and nephrology departments and in 52 healthy controls with SU <7 mg/dL recruited from among the hospitals' staff and patients' relatives.

The study was conducted according to the Declaration of Helsinki. Ethical approval for the study was obtained from both the Instituto Nacional de Cardiología and Instituto Nacional de Rehabilitación (Mexico City, Mexico) and informed consent was obtained from all participants.

### Clinical and laboratory assessments

All individuals underwent a detailed evaluation, including clinical history, musculoskeletal examination and laboratory testing. All study participants were asked to recall whether any musculoskeletal symptom had ever occurred. Both hyperuricemic subjects and controls underwent a clinical examination by an expert clinical rheumatologist, who recorded swelling and tenderness elicited by pressure, mobilization and contraction against resistance of the corresponding areas of study, to confirm the absence of musculoskeletal involvement. Hyperuricemic subjects without any clinical evidence of arthritis or enthesopathy underwent an US examination. Exclusion criteria were age <18 years, current or previous use of urate-lowering agents and nonsteroidal anti-inflammatory drugs or corticosteroids, malignancy or other concomitant rheumatic inflammatory conditions.

Fasting blood samples were collected from all individuals no longer than 3 days prior to US evaluation. SU concentrations were measured in duplicate by spectrophotometry (Hitachi 902; Roche Diagnostics, Indianapolis, IN, USA), mean values were used for analyses, and the results were expressed in milligrams per deciliter.

### US assessment

US examinations were performed using MyLab25 (Esaote Biomedica, Genoa, Italy) equipped with a 6- to 18-MHz broadband linear transducer. All patients were scanned by two rheumatologists (CP and CH-D) trained in musculoskeletal US. Representative images were acquired and digitally recorded, and an electronic file was created for each patient and used to complete a standardized *pro forma *offline file. Consensus between sonographers was obtained before the beginning of the study on both the scanning technique to adopt and the sonographic findings reported in the standardized *pro forma *file by scanning different individuals not involved in the present study. Discrepancies between sonographers were found in fewer than 10% of the images and were resolved by consensus.

The following anatomical areas were bilaterally scanned: knee (suprapatellar pouch, quadriceps tendon insertion, proximal and distal patellar tendon insertions and femoral hyaline cartilage), ankle (tibiotalar joint, posterior tibialis, peroneus longus and brevis tendons, as well as Achilles tendon) and first MTPJs (synovial membrane and hyaline cartilage).

All of the US examinations were performed using a multiplanar technique in accordance with the European League Against Rheumatism guidelines for musculoskeletal ultrasound in rheumatology [14]. Dynamic examination with flexion-extension was carried out to investigate the superficial margin of the hyaline cartilage in the first MTPJs.

US examination of the first MTPJs and tibiotalar joints, peroneus longus and brevis as well as posterior tibialis tendons were performed with the patient in a supine position with the knee in flexion (30°). The supine position with extended lower limbs was adopted for the quadriceps and patellar tendons and the suprapatellar pouch, while the Achilles tendons were examined while the patient was lying prone with the feet hanging over the edge of the examination table in flexion (90°).

Each anatomical area was scanned in gray scale mode to detect morphostructural changes and subsequently with the PD technique to detect abnormal blood flow. Blood flow was examined with a pulse repetition frequency of 750 KHz and a Doppler frequency between 6 and 8 MHz. Attention was given not to compress the tissues under examination to avoid a "blanching" of the PD signal due to the transducer pressure.

### US interpretation

US definitions described by the Outcome Measures in Rheumatoid Arthritis Clinical Trials (OMERACT) Special Interest Group were adopted for the study [15]. Joint effusion was recorded when anechoic or hypoechoic joint cavity widening was detected, while synovial hypertrophy was recognized as the presence of abnormal hypo- or hyperechoic tissue within the joint cavity. Additionally, hyperechoic enhancement of the superficial margin of hyaline cartilage was regarded as a surrogate of MSU crystal deposition (double contour sign), whereas inhomogeneous tendon and/or entheseal thickening and intratendinous hyperechoic bands defined the presence of enthesopathy or tendinopathy. Erosion was defined as a definite cortical interruption with a step-down contour defect in both longitudinal and transverse views. Enthesophyte was defined as a step-up prominence at the end of a normal bone profile.

### Statistical analysis

Frequencies (expressed as percentages) were used to describe categorical data and compared using the χ^2 ^test. Means (± SD) were used for continuous variables, and differences were assessed by unpaired *t*-tests. Correlations were evaluated by using the Spearman's coefficient (*r*_s_), while odds ratios (OR) were used to weigh the associations between hyperuricemia and US findings. *P *< 0.05 was considered significant. All analyses were two-tailed and were performed using GraphPad Prism 4.02 software (GraphPad Software, San Diego, CA, USA).

## Results

In all, 50 individuals with hyperuricemia and 52 controls with normouricemia were studied. Table 1 shows the demographic and clinical characteristics of the participants. Of note, both the mean age and the frequency of diseases associated with the metabolic syndrome were higher in hyperuricemic patients than in normouricemic individuals.

**Table 1:** Clinical and demographic data of the study populations

Parameter	Hyperuricemic (*n *= 50)	Normouricemic (*n *= 52)	*P *value
Mean age ± SD, yr	55.7 ± 16.6	47.3 ± 10.9	0.003
Male gender, *n *(%)	33 (66%)	35 (67%)	NS
Serum urate, mg/dL (mean ± SD)	8.1 ± 0.9	5.47 ± 0.90	<0.0001
Associated diseases			
Hypertension, *n*	24 (48%)	0	<0.0001
Diabetes mellitus, *n*	13 (26%)	0	<0.0001
Hyperlipidemia, *n*	8 (16%)	0	0.002

SD, standard deviation; NS, not significant.

A total of 100 knees, ankles and first MTPJs in patients with hyperuricemia and 104 in the normouricemic controls were studied. Seven (7%) of 100 knees from hyperuricemic patients showed joint cavity widening, whereas only two knees (1.9%; *P *= NS) from healthy controls revealed this abnormality. The proportion of joint cavity widening in the first MTPJs was 52% in hyperuricemic patients compared with 24% in the normouricemic group (*P *< 0.0001) (Table 2).

**Table 2:** Pathological ultrasonographic findings in joints^a^

Anatomical site and US findings	Hyperuricemic (*n *= 100 joints)	Normouricemic (*n *= 104 joints)	*P *value
First MTP joint, *n *(%)			
Double contour sign	25 (25%)	0	<0.0001
Joint cavity widening (synovial fluid/hypertrophy)	52 (52%)	25 (24%)	<0.0001
Power Doppler signal	0	0	NS
Bone erosion	12 (12%)	6 (5.7%)	NS
Knee, *n *(%)			
Double contour sign (femoral hyaline cartilage)	17 (17%)	0	<0.0001
Joint cavity widening (synovial fluid/hypertrophy)	7 (7%)	2 (1.9%)	NS

^a^US, ultrasonographic; MTP, metatarsal-phalangeal joint; NS, not significant.

**Figure 1:**
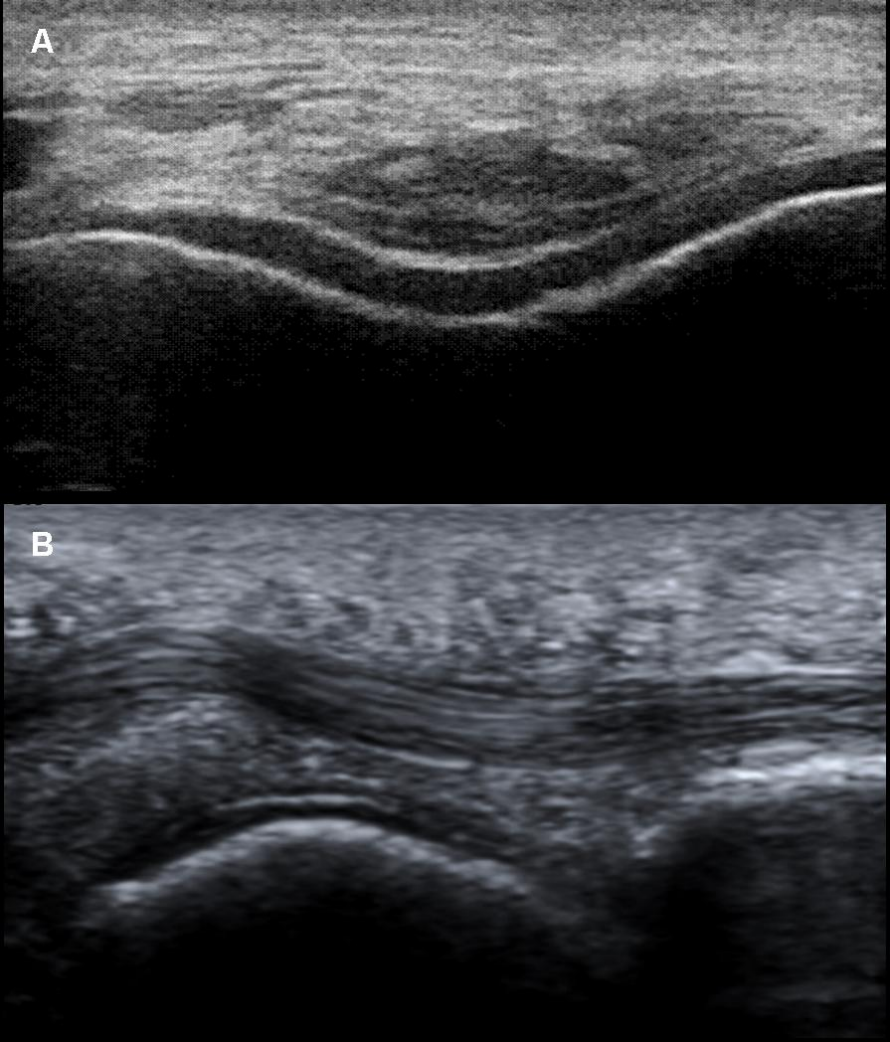
**The double contour sign**. Hyperechoic enhancement of the chondrosynovial interface secondary to the monosodium urate crystal deposition. **(A) **Transversal scan of the femoral cartilage surface. **(B) **Longitudinal view of the plantar aspect in the first metatarsal-phalangeal joint.

On femoral hyaline cartilage, the double contour sign (Figure 1A) was present in 17 of 100 knees from hyperuricemic patients in contrast to none in the control group (*P *< 0.0001), giving an odds ratio (OR) of 43.8 (95% confidence interval (95% CI), 2.9 to 739). The prevalence of the double contour sign in the first MTPJs (Figure 1B) was also higher in hyperuricemic patients (25% vs. 0%; *P *< 0.0001), with an OR of 34.3 (95% CI, 4.5 to 259). However, no correlation between SU concentration and the presence of the double contour sign was found (*r*_s _-0.06; 95% CI, -0.3 to 0.2).

Tophi formation was found in 18 individuals with hyperuricemia but in none of the normouricemic controls (*P *< 0.0001), producing an OR of 46.8 (95% CI, 2.7 to 789); however, mean SU concentrations were similar regardless the presence of tophi (8.13 ± 0.89 vs. 8.13 ± 0.99 mg/dL; *P *= NS). Intra-articular tophi were found in eight (8%) hyperuricemic patients but in none of the normouricemic controls (*P *= 0.003).

Tendon examinations showed patellar enthesopathy (12% vs. 2.9%; *P *= 0.01) and intratendinous tophi (6% vs. 0; *P *= 0.01), as well as Achilles enthesopathy (15% vs. 1.9%; *P *= 0.0007), to be more frequent in hyperuricemic patients than in normouricemic individuals (Table 3). Tenosynovitis was found only in three hyperuricemic patients (two in the peroneus longus tendon and one in the posterior tibialis tendon). No PD signal was found in any anatomical area examined.

**Table 3:** Pathological ultrasonographic findings in tendons^a^

Anatomical site	US finding	Hyperuricemic (*n *= 100 tendons)	Normouricemic (*n *= 104 tendons)	*P *value
Quadriceps tendon, *n *(%)	Enthesopathy	5 (5%)	5 (4.8%)	NS
	Tophus	2 (2%)	0	NS
Patellar tendon	Enthesopathy	12 (12%)	3 (2.9%)	0.01
(proximal/distal), *n *(%)	Tophus	6 (6%)	0	0.01
Posterior tibialis tendon	Enthesopathy	0	0	NS
	Tenosynovitis	1 (1%)	0	NS
	Tophus	0	0	NS
Peroneus tendon	Enthesopathy	0	0	NS
(brevis/longus), *n *(%)	Tenosynovitis	2 (2%)	0	NS
	Tophus	0	0	NS
Achilles tendon, *n *(%)	Enthesopathy	15 (15%)	2 (1.9%)	0.0007
	Tophus	2 (2%)	0	NS

^a^US, ultrasonographic; NS, not significant.

## Discussion

The present study was aimed at demonstrating a wide spectrum of subclinical structural damage in asymptomatic individuals with hyperuricemia. Our results support the hypothesis that the morphostructural changes suggestive of gouty arthritis induced by hyperuricemia can occur in both intra- and extra-articular structures in asymptomatic individuals.

Chronically elevated SU has not usually been considered to play a pathogenic role in tissue damage. However, large prospective population studies are challenging this notion, since they have found that SU levels are reliable and consistent predictors of progression for endothelial dysfunction [16], coronary artery disease [17,18] and renal failure [2]. In this line of thought, our results support the existence of both intra- and extra-articular tissue damage caused by the persistent elevation of SU [5]. The presence of MSU crystals in the synovial fluid from asymptomatic individuals with hyperuricemia has been demonstrated on the basis of polarized light microscopy since the early 1980s [4]. In accord with this evidence, the double contour sign has been described solely in gout and seems to represent the preference of SU to crystallize on the surface of cartilage [7,11,19]. Even when the underlying mechanisms for this preference need further clarification, it has been shown that the normal components of cartilage chondroitin sulfate and phosphatidylcholine facilitate the nucleation and subsequent crystallization of MSU [20]. As confirmation of the presence of MSU in the hyaline cartilage, Thiele and Schlesinger [21] recently demonstrated the disappearance of the double contour sign in patients with gout successfully treated with urate-lowering agents who had maintained SU levels below 6 mg/dL for at least 7 months. Also, tophi formation detected in our study further confirmed the presence of MSU crystal tissue deposition in both intra- and extra-articular structures from asymptomatic hyperuricemic individuals as previously suggested [5]. This may strengthen the need for treatment necessity in asymptomatic individuals with hyperuricemia and indisputable US features of MSU crystal tissue deposition such as the double contour sign or the presence of tophi [3].

Recently, US has been shown to be of value in revealing subclinical joint and tendon inflammation in patients with other inflammatory conditions such as arthritis [22,23], psoriasis [24] and Sjögren's syndrome [25]. This prompted us to investigate its ability to identify the involvement of the hyaline cartilage, tendons and joints in individuals with asymptomatic hyperuricemia and no signs of inflammation or musculoskeletal complaints. Our results are in agreement with a previous report [5]. Indeed, Puig *et al. *[5] studied 35 asymptomatic individuals with hyperuricemia and found tophi formation in both tendons and synovium in 34% of patients, with a special preference for the distal patellar tendon. Here we have extended the US evaluation to other anatomical sites characteristically involved in gout, such as the hyaline cartilage and first MTPJs, and have shown that MSU crystal deposition and structural damage in these locations may be even greater and more frequent than in tendons. Additionally, the present study was carried out in a higher number of participants and included a normouricemic, healthy control group. Further differences between studies are related to our findings of bone erosion and synovial fluid and/or hypertrophy, both of which are common features of gout but were not assessed in the study by Puig *et al. *[5].

We are aware that our study has limitations, including the lack of validation for morphostructural changes by other techniques, the absence of longitudinal follow-up to determine the predictive value of US in the development of established gout, the lack of MSU crystal diagnosis in those patients with US changes suggestive of gouty arthritis, and the low sensitivity of the PD used. Furthermore, the sonographers were not blinded to whether they were examining a hyperuricemic individual or a control. Finally, the demographic differences between study groups, namely, younger healthy participants, cannot be ignored.

The ability of US to detect signs of subclinical gout should be the object of longitudinal investigations. Ongoing follow-up of the patients recruited in the present study will provide further information regarding the predictive value of US findings for the development of gouty arthritis.

## Conclusions

Our US findings open a new battlefront in the current debate about the use of urate-lowering agents in asymptomatic patients with persistent hyperuricemia. Also, these findings support musculoskeletal US as a useful, noninvasive tool to detect anatomical damage in the hyaline cartilage, synovial tissue and tendons of asymptomatic individuals with hyperuricemia.

## Abbreviations

MSU: monosodium urate; MTPJs: metatarsal-phalangeal joints; OR: odds ratio; PD: power Doppler; SU: serum urate; US: ultrasound.

## Competing interests

The authors declare that they have no competing interests.

## Authors' contributions

CP participated in the conception of study and interpretation of data, was involved in drafting the manuscript, and gave final approval of the version of the paper to be published. LMA-G participated in the design of the study and the acquisition and interpretation of data and performed the statistical analysis and the drafting of the manuscript. CS, PR-H, CH-D and FH participated in the acquisition of data, performed the clinical examinations and carried out the ultrasound studies. AV made substantial contributions to the conception and design of the study, participated in the acquisition of data, and helped to draft the manuscript. MW participated in the acquisition of data and was involved in revising the manuscript for important intellectual content. All authors read and approved the final manuscript.
